# Investigating the association between season of birth and symptoms of depression and anxiety in adults

**DOI:** 10.1371/journal.pmen.0000296

**Published:** 2025-07-30

**Authors:** Arshdeep Kaur, Mikael Mokkonen, Cayley E. Velazquez

**Affiliations:** Department of Biological and Health Sciences, Kwantlen Polytechnic University, Surrey, British Columbia, Canada; PLOS: Public Library of Science, UNITED KINGDOM OF GREAT BRITAIN AND NORTHERN IRELAND

## Abstract

The season of birth exposes a fetus to varying environmental and developmental conditions which may influence health outcomes after birth. The influence of season of birth has been observed in neuropsychiatric disorders and chronic health conditions. However, research on the association between season of birth and common mental health disorders is currently limited. This global study sought to fill this gap by investigating the association between season of birth and symptoms of depression and anxiety in adults using a survey-based cross-sectional research design. Participants for this study (n = 303) were primarily women (65%) with a mean age of 26 years old. Season of birth was assessed as Winter, Spring, Summer or Fall based on birth month. Depression symptoms were assessed using the Patient Health Questionnaire-9 (PHQ-9) and anxiety symptoms were assessed using the Generalized Anxiety Disorder-7 (GAD-7) scale. A generalised linear mixed model was used to assess the association of season of birth with symptoms of depression and anxiety, by sex, controlling for age, income, and latitude differences. Mental health conditions were common among participants, with 84% and 66% of participants experiencing symptoms of depression and anxiety, respectively. Season of birth was not associated with anxiety symptoms However, while males born during summer had a higher risk of developing depression symptoms, there was no observed association among females. This study provided further evidence supporting the association between season of birth and emergence of adulthood depression symptoms, particularly in relation to sex. Future studies should investigate the biological sex-specific mechanisms that underlie mental health outcomes in relation to the developmental conditions experienced at different times of the year.

## 1. Introduction

Mental health has profound implications for daily life, thus exploring its contributing factors can reveal ways to improve quality of life. Mental health is described as a state of well-being in which individuals can cope effectively with typical stresses of life, live productively, and contribute to the community [1]. Mental health disorders significantly contribute to the increasing health burden worldwide [2]. According to the Global Burden of Diseases, Injuries, and Risk Factors Study, one in every eight people had a mental disorder in 2019 [2]. Specifically, anxiety and depression serve as two of the most common mental disorders [3]. In addition, these conditions have also been linked to an increased risk of developing physical comorbidities and acquiring multiple chronic health conditions [4]. Moreover, lost productivity due to depression and anxiety disorders cost the global economy approximately $1 trillion USD each year [3]. Therefore, it is important to investigate and identify the risk factors associated with depression and anxiety symptoms to alleviate the burden of their onset.

Mental health is shaped by a multitude of biological, social, behavioural, and developmental factors [5]. In particular, social determinants of health, such as safe housing, secure employment, food security, and educational attainment have been shown to strongly influence mental health outcomes [5]. Similarly, key demographic characteristics such as age, sex, race, and ethnicity have also been significantly associated with mental health outcomes [5]. However, there has been limited research into how early life exposures or developmental factors impact mental health outcomes in adulthood. Given that the prenatal and early postnatal stages of life are critical developmental periods, season of birth serves as a useful indicator of various environmental factors at play during these early stages. These factors may include nutrient availability, environmental chemicals, drugs, infections and other stressors. Furthermore, environmental factors linked to season of birth can vary notably between the Northern and Southern Hemispheres, reflecting differences in photoperiod, climatic conditions, maternal nutrition, and the seasonal prevalence of infections and allergens [6]. Furthermore, the evolving impact of global climate change, including alterations in temperature, infectious disease cycles, and food availability, may influence perinatal environmental exposures in complex ways. Seasonality shapes many aspects of the environment which then affects human health [7]. Historically, changes in environmental conditions throughout the year have influenced resource availability, migration patterns, and psychological conditions in the human population [8]. The concept of “birth season effects” recognizes that in-utero conditions of a fetus can exert long-lasting impacts on health and that such conditions can be altered by seasonal influences [8].

The relationship between season of birth and health may be attributed to adaptive developmental plasticity mechanisms that occur during early developmental periods when an individual is highly sensitive to their environment [9]. Accordingly, fetal exposure to certain environmental conditions can result in irreversible programming of tissues in a manner that predisposes individuals to later diseases in life [10]. The “Fetal Origins of Adult Disease” (FOAD) hypothesis proposes that parameters of early growth such as low birth weight can influence one’s risk of developing chronic diseases including cardiovascular disease and type 2 diabetes [11]. More recently, a large-scale research study (Season-Wide Association Study) which used a hypothesis-free algorithm with 1,688 conditions as initial input revealed 55 conditions associated with birth month.) [12]. The Season-Wide Association study confirmed many connections between birth month and disease including asthma, respiratory syncytial virus, and Attention-Deficit Hyperactivity Disorder (ADHD) [12]. Such findings support growing evidence that the risk of developing certain diseases can be traced back to patterns of early development.

The study of the association between season of birth and development of mental health disorders has largely focused on severe psychiatric disorders, particularly schizophrenia [13]. Previous work has shown an elevated risk of developing schizophrenia for individuals born in winter and spring months, specifically for those born between December and March in the Northern Hemisphere [13–15]. The risk of developing mental health disorders may increase with birth during specific seasons, possibly due to seasonal variation in nutrient supply, exposure to infectious agents, and sunlight [16]. Other mental health disorders which have been associated with season of birth include bipolar disorder, suicidal behavior, and schizoaffective disorder [13]. Specifically, the association between season of birth as a possible risk factor for depression was first postulated in the early 20th century [8], but findings associating the occurrence of mood disorders with birth seasons have also been inconsistent [16–22].

Differences in disease risk by sex have been observed in other studies associating season of birth with physical health outcomes (e.g., multiple sclerosis) [22]. It is possible that unaccounted for sex differences between birth season and mental health have played a role in the inconsistent findings to date. Because the relationship between early life factors and mental health risk is far less explored, the aim of this study was to further investigate the association between season of birth and symptoms of depression and anxiety in adults using a survey-based cross-sectional research design, accounting for biological sex, and controlling for other demographic variables such as age and socio-economic status.

## 2. Materials and methods

### 2.1 Ethics statement

This study was approved by the Multi-Jurisdictional Board of Record in accordance with the Tri-Council Policy Statement: Ethical Conduct for Research Involving Humans (TCPS2). An ethical approval certificate (H23-03980) for a harmonized minimal risk behavioural study was issued by the Research Ethics Board of Kwantlen Polytechnic University. Study participants were not involved in the design, conduct, reporting, or dissemination plans of this research.

### 2.2 Study design and participants

A survey-based cross-sectional research design was used to investigate the association between season of birth and mental health outcomes. Inclusion criteria required participants to be 18 years old or older. Participants were recruited from January 26 to March 10, 2024 through multiple channels, including social media platforms, in-person networking, and via the placement of recruitment posters. Social media recruitment took place using the researchers’ personal accounts and university-affiliated clubs’ accounts on platforms such as Instagram, Facebook, and Discord. Recruitment posters were posted on bulletin boards across different universities across Vancouver, BC, Canada. In addition, participants were offered incentives; specifically, the opportunity to win electronic Amazon Canada gift cards valued at $50.

The survey was conducted online between January and March, 2024, using the internet based SurveyMonkey platform. Online informed consent was gathered prior to survey completion. The survey consisted of four sections: demographics, health behaviours, health outcomes, and birth, early life, and family history, and took approximately 20 minutes to complete. In total, 424 responses were collected, however, following data cleaning, 121 participants were removed due to poor response quality, straight-line bias, duplicate entries, or extremely rapid completion time. In addition, participants were automatically excluded if they only provided demographic information.

### 2.3 Measures

#### 2.3.1 Independent variable.

Season of birth was determined by grouping participant birth months into their respective seasons: winter (December, January, February), spring (March, April, May), summer (June, July, August) and fall (September, October, November).

#### 2.3.2 Dependent variables.

Depression symptoms were assessed using the PHQ-9, a nine-item scale [23]. Participants indicated how often they experienced each depression symptom in the last two weeks on a scale of “not at all”, “several days”, “more than half the days” and “nearly every day”, with scores ranging from 0 (not at all) to 3 (nearly every day). Scores from all nine items were summed, with a total score ranging from 0 to 27. Higher scores indicated a greater severity of depression symptoms.

Anxiety symptoms were assessed using the GAD-7, a seven-item scale [24]. Participants indicated how often they were bothered by different symptoms of anxiety during the last two weeks on a scale of “not at all”, “several days”, “more than half the days” and “nearly every day”, with scores ranging from 0 (not at all) to 3 (nearly every day). Scores from all seven items were summed, with a total score ranging from 0 to 21. Higher scores indicated a greater severity of anxiety symptoms.

For both the PHQ-9 and GAD-7 scales, a threshold cut-off score of 10, guided by previous research [25,26], was used to classify the presence or absence of depression and anxiety symptoms, respectively.

#### 2.3.3 Control variables.

Age, income, and geography were used to control for confounding effects in the model that assessed depression and anxiety scores among individuals. Mental health is influenced by age at various phases of life, e.g., children, young adults, and the elderly. Socioeconomic factors, mainly income, play a significant role in mental health outcomes. Lastly, latitude or geographic location also influence mental health outcomes through environmental factors such as climate, exposure to sunlight, and precipitation [6–8].

### 2.4 Statistical analysis

Descriptive analyses (mean ± standard error (SE), or frequency (n, %)) were conducted to illustrate the distribution of participants by season of birth, sociodemographic variables, and mean scores of depression and anxiety symptoms. A chi-square test of independence on covariates including sex, age, and income group was performed to ensure that any observed associations were not attributable to variations in subgroup composition. Generalized Linear Mixed Model (GLMM) analysis was performed to determine the association between season of birth and symptoms of depression and symptoms of anxiety (two separate models were run independently) [25]. The GLMM analysis accounted for the potential influence of individual characteristics and additional covariates. It also allowed for the examination of both fixed and random effects on the presence of depression and anxiety symptoms. The fixed effects which consistently impact depression and anxiety symptoms across all individuals included season of birth and sex at birth. The random effects which could account for the variability between individuals included age, geography, and socioeconomic factors. The effect of geographical variation on the month of birth was controlled by using latitude coordinates. In the analysis of season of birth in relation to severity of depression and anxiety symptoms, a generalised linear mixed model (GLMM) wherein “sex” “season of birth” and their interaction were the fixed effects and “income”, “age”, and interaction of “month of birth” and “latitude” were the random effects. The Akaike information criterion (AIC) was used to select the best fit models in the GLMM analyses [25]. All statistical analyses were conducted using Statistical Package for the Social Sciences (SPSS) version 29 software.

## 3. Results

Participants for this study (n = 303) were primarily women (65%) with a mean age of 26 years (0.5 years) (see Table 1). Most participants were between 18–29 years old (79.6%). Nearly half (40%) of participants had attained a high school diploma as their highest level of education. Participants were primarily of South Asian (31.7%), White (24.4%), and Filipino (15.2%) descent. Mental health conditions were common among participants, with 84% and 66% of participants experiencing symptoms of depression and anxiety, respectively.

**Table 1:** Demographic characteristics of participants with mean GAD-7 and PHQ-9 Score (n = 303).

Characteristic	Frequency (n, %)	GAD-7 Score (Mean ± S.E.)	PHQ-9 Score (Mean ± S.E.)
*Sex at birth*			
Male	106 (35%)	10·63 ± 0·33	**12·93 ± 0·41**
Female	197 (65%)	**11·22 ± 0·24**	**13·36 ± 0·29**
*Age (years)*			
18-29	241(79·6%)	**11·11 ± 0·22**	**13·24 ± 0·26**
30-40	39 (12·9%)	**10·94 ± 0·53**	**14.18 ± 0.58**
Above 40	23 (7·6%)	10·11 ± 0·82	11·29 ± 0·89
*Race/Ethnicity*			
Indigenous	3 (1·0%)	10·33 ± 0·88	**15·67 ± 2·33**
Black	8 (2·6%)	11·00 ± 0·90	12·86 ± 1·53
Chinese	23 (7·6%)	10·09 ± 0·57	**13·15 ± 0·82**
Filipino	46 (15·2%)	**11·21 ± 0·60**	**13·07 ± 0·72**
Central Asian	1 (0·30%)	**10·00 ± 0·00**	**15·00 ± 0·00**
Korean	5 (1·7%)	12·40 ± 1·78	10·80 ± 1·88
Latin, Central, South American	9 (3·0%)	11·00 ± 0·96	**12·22 ± 0·89**
South Asian	96 (31·7%)	**11·71 ± 0·33**	**13·37 ± 0·42**
Southeast Asian	5 (1·7%)	8·80 ± 1·83	**14·60 ± 1·54**
West Asian	7 (2·3%)	10·57 ± 1·40	**13·17 ± 1·22**
White	74 (24·4%)	10·55 ± 0·38	**13·22 ± 0·44**
Multiracial	24 (7·9%)	10·91 ± 0·72	**13·70 ± 0·80**
Unspecified	2 (0·7%)	NA	NA
*Highest Education Level*			
Less than high school	4 (1·3%)	11·25 ± 1·55	12·50 ± 1·94
Highschool degree	122 (40·3%)	**11·31 ± 0·30**	**13·33 ± 0·38**
Trade certificate or diploma	10 (3·3%)	10·11 ± 0·75	12·22 ± 1·85
College certificate, diploma	31 (10·2%)	11·07 ± 0·79	**13·39 ± 0·86**
University certificate, diploma	37 (12·2%)	11·17 ± 0·60	**13·42 ± 0·77**
Bachelor’s degree	73 (24·1%)	**10·96 ± 0·37**	**13·19 ± 0·41**
Graduate degree	26 (8·6%)	9·77 ± 0·60	**12·65 ± 0·59**
*Annual Household Income*			
Less than $15,000	71 (23·4%)	1**0·94 ± 0·43**	**13·22 ± 0·49**
$15,000-24,999	37 (12·2%)	**11·66 ± 0·45**	**13·26 ± 0·73**
$25,000-$34,999	21 (6·9%)	10·33 ± 0·77	**13·50 ± 1·16**
$35,000-$49,999	23 (7·6%)	10·61 ± 0·70	**13·17 ± 0·77**
$50,000-$74,999	25 (8·3%)	10·71 ± 0·61	**12·14 ± 0·42**
$75,000- $99,999	21 (6·9%)	11·33 ± 0·90	**13·22 ± 0·79**
$100,000 and more	20 (6·6%)	10·89 ± 0·90	**13·30 ± 0·92**
Unspecified	85 (28·1%)	NA	NA
*Occupational Status*			
Full time	76 (25·1%)	10·55 ± 0·40	**12·82 ± 0·40**
Part time	28 (9·2%)	**11·11 ± 0·58**	**13·54 ± 0·81**
Homemaker	2 (0·7%)	10·50 ± 3·5	**13·50 ± 1·50**
Student	121(39·9%)	**11·08 ± 0·30**	**13·29 ± 0·40**
Employed Full Time & Student	9 (3·0%)	10·67 ± 1·35	**14·00 ± 1·20**
Employed Part Time & Student	49 (16·2%)	**11·70 ± 0·49**	**13·14 ± 0·58**
Retired	2 (0·7%)	10·00 ± 1·0	10.00 ± 1·00
Unspecified	16 (5·3%)	NA	NA
*Relationship Status*			
Single	147 (48·5%)	**11·33 ± 0·27**	**13·35 ± 0·36**
In a relationship	99 (32·7%)	**10·86 ± 0·35**	**13·34 ± 0·39**
Married	46 (15·2%)	10·40 ± 0·57	**12·37 ± 0·57**
Divorced or separated	6 (2·0%)	10·40 ± 0·87	**13·20 ± 0·80**
Widowed	1 (0·3%)	7·00 ± 0·00	8·00 ± 0·00
Unspecified	4 (1·3%)	NA	NA

*Note*: Bold values are significantly above the cut-off score of 10, indicating the group is symptomatic for anxiety (GAD) or depression (PHQ).

Most demographic groups had mean scores (mean ± S.E.) for symptoms of depression and anxiety significantly above the threshold cut-off (Table 1). Participants in the age group of 18–29 years old had a mean score of 11.11 ± 0.22 for anxiety and 13.24 ± 0.26 for depression symptoms, whereas those in the age group of 30–40 had a mean score of 10.94 ± 0.53 for anxiety and 14.18 ± 0.58 for depression symptoms. Generally, females exhibited scores above the threshold cut-off score for both depression (13.36 ± 0.29) and anxiety symptoms (11.22 ± 0.24). Males generally exhibited scores above the threshold only for depression symptoms (12.93 ± 0.41). There was a pattern of significant depression and anxiety scores in the lower age and income groups compared to the higher age and income groups. Participants who were single or in a relationship also exhibited significantly higher depression and anxiety scores than participants who were married, divorced or widowed. However, no clear patterns for depression and anxiety scores were evident in terms of race/ethnicity, education level, or occupational status (Table 1).

The distribution of participant characteristics across seasons was not significantly different (Table 2). There was no significant association between season of birth and sex for females (chi-square; χ² = 4.157, df = 3, p = 0.245). Similarly, there was no significant association between season of birth and sex for males (chi-square: χ² = 3.962, df = 3, p = 0.266). There was also no significant association between season of birth and each of the respective age groups (chi-square: 18–29 years old: χ² = 2.469, df = 3, p = 0.481; 30–40 years old: χ² = 1.680, df = 3, P = 0.468; 40 and above: χ² = 1.333, df = 3, p = 0.199). For income group, there was no significant association in relation to season of birth, indicating a relatively even distribution of study participants across the categories.

**Table 2:** Covariates of age, sex, and income distributed by season of birth.

	Winter (n,%)	Spring (n,%)	Summer (n,%)	Fall (n,%)	*p*-value
*Age Group n = 303*					
18-29	62 (20·5%)	50 (16·50%)	66 (21·8)	63 (20·80%)	0·481
30-40	6 (2·0%)	10 (3·30%)	10 (3·30%)	13(4·30%)	0·468
Above 40	9 (3·0%)	2 (0·66)	7 (2·3%)	5 (1·70%)	0·199
*Sex n = 303*					
Male	33 (10·89%)	21 (6·93%)	30 (9·90%)	22 (7·26%)	0·266
Female	44 (14·52%)	41 (13·5%)	53 (17·5%)	59 (19·47%)	0·245
*Income Group n = 218*					
Less than $15,000	18 (8·26%)	14 (6·42%)	19 (8·72%)	20 (9·17%)	0·760
$15,000-24,999	14 (6·42)	5 (2·29)	9 (4·13%)	9 (4·13%)	0·221
$25,000-$34,999	5 (2·29%)	6 (2·75%)	5 (2·29%)	5 (2·29%)	0·986
$35,000-$49,999	4 (1·83%)	8 (3·67%)	9 (4·13%)	2 (0·92%)	0·127
$50,000-$74,999	7 (3·21%)	7 (3·21%)	6 (2·75%)	5 (2·29%)	0·932
$75,000- $99,999	5 (2·29%)	5 (2·29%)	5 (2·29%)	6 (2·75%)	0·986
$100,000 +	8 (3·67%)	3 (1·38%)	2 (0·92%)	7 (3·21%)	0·158

*Note:* A chi-square test was conducted to test each category against the 1:1:1:1 null hypothesis.

**Fig 1:**
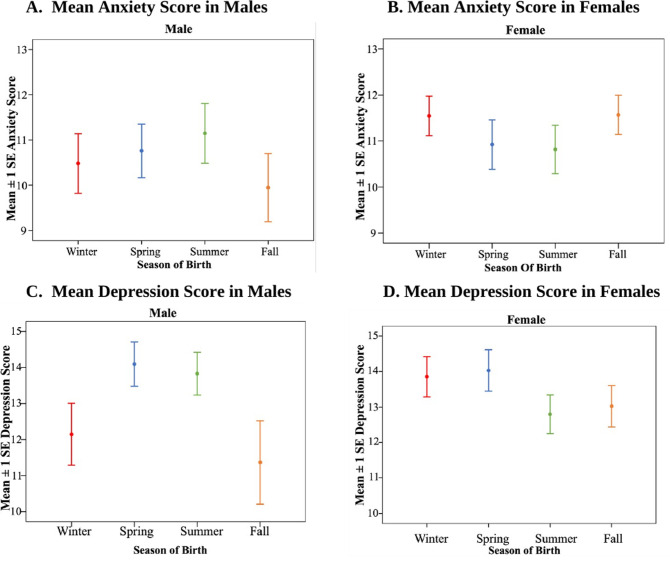
Mean anxiety and depression scores by season of birth, stratified by sex. **(A)** Mean anxiety score in males **(B)** Mean anxiety score in females **(C)** Mean depression score in males **(D)** Mean depression score in females. Note: Males and females do not significantly differ in their mean anxiety scores in relation to season of birth. Males were shown to have higher depression scores during the spring and summer seasons, while females were shown to have higher depression scores during the winter and spring seasons. The error bars represent means depression scores.

Overall, individuals born during the summer season had a greater likelihood of experiencing anxiety and depression symptoms within the sample (Tables 3 and 4). For both anxiety and depression, more participants in general reported having moderate symptoms than mild or severe symptoms. Interestingly, males born during the summer displayed higher PHQ-9 depression scores compared to males born during other seasons (Fig 1; GLMM: season of birth F_3,180_ = 1.896, P = 0.132; sex F_1,180_ = 1.896, P = 0.880; season of birth*sex F_3,180_ = 2.588, P = 0.054). No such association was observed for females. However, the best fit model for anxiety score revealed no significant association between season of birth and anxiety symptoms among males or females (GLMM: season of birth F_3,182_ = 0.317, P = 0.813; sex F_1,182_ = 0.930, P = 0.336; season of birth*sex F_3,182_ = 1.381, P = 0.250).

**Table 3:** Participant frequency distribution of anxiety severity by season of birth.

		Season of Birth				
		Winter	Spring	Summer	Fall	Total
Anxiety Severity	None to Minimal Anxiety	1	0	4	2	7
	Mild Anxiety	23	20	21	21	85
	Moderate Anxiety	37	34	42	40	153
	Severe Anxiety	8	6	9	7	30
Total		69	60	76	70	275

*Note:* None to minimal anxiety, and mild anxiety levels fall below the cut-off score of 10, indicating absence of anxiety symptoms according to the GAD-7 scale. 28 participants did not complete all required items on the GAD-7 anxiety scale, resulting in a reduced sample size of n = 275 for analyses.

**Table 4:** Participant frequency distribution of depression severity by season of birth.

		Season of Birth				
		Winter	Spring	Summer	Fall	Total
Depression Severity	None to Minimal Depression	2	0	1	2	5
	Mild Depression	9	5	9	13	36
	Moderate Depression	33	27	43	29	132
	Moderately Severe Depression	16	23	22	19	80
	Severe Depression	7	3	3	5	18
Total		67	58	78	68	271

*Note:* None to minimal depression, and mild depression levels fall below the cut-off score of 10, indicating absence of depression symptoms according to the PHQ-9 scale. 32 participants did not complete all required items on the PHQ-9 depression scale, resulting in a reduced sample size of n = 271 for analyses.

## 4. Discussion

While the influence of season of birth has been observed in several acute neuropsychiatric disorders (e.g., schizophrenia) and chronic health conditions (e.g., cardiovascular diseases), [12,13] this is one of the first studies to show sex-specificity in these season of birth effects on more common mental illnesses. Because season of birth serves as a proxy for the various environmental factors in prenatal and early post-natal life, it is crucial to understand its role in the development of prevalent mental health disorders which continue to be a major public health concern. In this study, there was no difference in the distribution of births by season in terms of sex, age group, and income level, demonstrating homogeneity across the covariates studied. Additionally, no clear patterns for depression and anxiety scores were evident in terms of race or ethnicity. However, the emergence of depression and anxiety symptoms was found to be more prevalent among specific vulnerable demographic groups. Consistent with previous literature, females and individuals in lower socioeconomic groups had higher severity of anxiety and depression symptoms, suggesting that sex and income are strongly associated with the emergence of mental health disorders [26,27].

In the present study, season of birth was not directly associated with depression symptoms. However, there was a marginally significant interaction between season of birth and sex with respect to depression symptoms (Fig 1). Specifically, males born during the summer season were at greater risk of experiencing depression symptoms. Previous research has found that individuals born in summer have a higher likelihood of depression symptoms [19]; however, no differences based on sex were reported. Moreover, males experienced higher levels of depression if born during the spring and summer seasons, whereas females had higher levels if born during the winter and spring seasons (Fig 1). This finding is in line with a Korean study that found patients born in spring and summer had more severe clinical symptoms of depression than those born in fall and winter [28]. These findings suggest a seasonal variation in the severity of depression symptoms, with both males and females experiencing higher severity if born during the spring months. As evidence is very limited on sex-based differences, it is notable that we found this difference between males and females with respect to depression symptoms. A possible explanation is that environmental exposures associated with different seasons such as variations in photoperiod, maternal nutrition, and seasonal infections may influence neurodevelopment and contribute to sex-specific vulnerability to psychiatric disorders. A previous study provides evidence for this, showing that season of birth affects gray matter volume in the left superior temporal sulcus differently in males and females. Men born in fall and winter showed increased gray matter in the left superior temporal sulcus compared to those born in spring and summer, while women demonstrated the inverse pattern [29]. The superior temporal sulcus is important in social cognition, auditory processing, and emotion perception, all of which are commonly disrupted in mood disorders. Altered development in this region may affect social-emotional regulation, potentially influencing vulnerability to depressive and anxiety symptoms in a sex-dependent manner [29]. This interaction between sex and season of birth highlights the complex interplay between biological and environmental factors in adult mental health outcomes.

Season of birth had no significant association with symptoms of anxiety (Fig 1). There was also no significant interaction effect between variables of sex and season of birth, indicating a lack of association altogether. This finding is consistent with recent research which found no significant association between participants’ season of birth and symptoms of depression or anxiety [20]. The researchers suggested that improvements in nutritional access and prenatal education over the years could potentially explain the lack of association between season of birth and mental health symptoms in the study [20]. Nevertheless, our current study found a significant association between season of birth and depression symptoms, possibly due to differences in sample demographics. Given the uneven distribution of biological sex and age in the dataset, these findings should be interpreted with caution when generalizing results. This discrepancy in findings may be attributable to one of the limitations reported by Csajbók, Kagstrom, and Cermakova [20], specifically its lack of generalizability owing to a sample that is mentally healthier than the broader population [20]. Therefore, it would be important to assess these patterns with a larger sample of the population, as greater statistical power may potentially reveal further or differing effects.

The PHQ-9 and GAD-7 scales, highly reliable and validated measurement tools used in this research, add to the validity of the results [23,24]. Both these scales have sound psychometric properties and have been widely used in primary care settings and in the general population. Most importantly, these scales were developed based on the DSM-V diagnostic criteria for depression and generalized anxiety disorder. However, this study is not without limitations. First, using a cross-sectional design means that causation between season of birth and mental health can only be inferred. Second, data on abiotic factors (e.g., sunlight exposure, temperature, allergens, pollution, and precipitation rates) were not accessible and could not be incorporated into the analyses. Moreover, data collection in this study spanned a period of only two months, so it may not have captured variations in depression and anxiety scores. The Canadian Community Health Survey (CCHS) data collected in 2015 and 2016 revealed seasonal variations in the occurrence of depression symptoms, with symptoms being more frequently reported in the winter months [30]. As data in this study was collected during February and March (winter months), more participants may have been susceptible to symptoms of depression. However, our experimental design controlled for seasonal fluctuations by focusing on the time of year when people are most susceptible to neuropsychiatric symptoms. Furthermore, this dataset included a substantial percentage of students (39.9%) (Table 4). A national study of Canadian post-secondary students revealed that 83.7% and 86% of students experienced symptoms of anxiety and depression, respectively [31]. Despite these limitations, the current findings provide valuable insights into the relationship between season of birth and common mental health disorders, demonstrating that interventions and policy considerations should account for the potential influence of birth season (and sex differences) on mental health outcomes.

It was noteworthy that a large proportion of the sample scored above the threshold for diagnosing depression and anxiety symptoms (Table 1). The prevalence of these mood disorders within the study cohort, while high, aligns with general nationwide data [32]. In a 2022 survey conducted by the Centre for Addiction and Mental Health (CAMH), 25.1% of Canadians experienced moderate to severe anxiety, while 22.3% experienced feelings of depression [32]. This study found that a majority of participants, specifically 66% and 84%, reported symptoms of anxiety and depression, respectively. Further work would need to be done to validate whether any kind of bias, such as digital access or motivational bias, may have confounded these results. Nevertheless*,* these findings highlight the importance of addressing mental health disorders within the larger population, particularly among younger adults who may be at particular risk. These findings have significant implications for public health policy and relevant academic and/or governmental authorities to prioritize mental health needs in policymaking decisions.

This study offers further evidence supporting the association of season of birth with depression symptoms among adults. The potential influence of season of birth has been explained through factors such as maternal nutrition, seasonal infectious diseases, genetics, and hormone levels [8,19]. However, given the variability of findings reported across the literature, multiple mechanisms specific to geography, environment, genetics, and social factors may mediate observed associations in relation to season of birth [16–21]. This study provides new knowledge about the sex-specific interaction between season of birth and male depression scores. These findings offer valuable insights for guiding and informing targeted mental health interventions and policies that address the unique needs of different demographic groups. However, the underlying mechanisms of how birth seasonality relate with mental health outcomes in adulthood remain unclear. Future research could delve into biological experiments and genetic research to further understand the impact of season of birth and its underlying biological mechanisms. Future research could also aim to build a more comprehensive model accounting for life history characteristics (e.g., early childhood experiences, assortative mating patterns), behavioural (e.g., maternal supplement use) and environmental determinants (e.g., daylength, precipitation rate) that can potentially provide further insights into the patterns that may influence season of birth effects.
